# Behcet's disease in an adult male from Nepal: A case report

**DOI:** 10.1002/ccr3.4912

**Published:** 2021-10-13

**Authors:** Madan Basnet, Kamal Gautam, Bishnu Deep Pathak, Abisha Phudong, Suman Gaire, Narayan Bohara, Ayushi Srivastava

**Affiliations:** ^1^ Tribhuvan University Teaching Hospital Kathmandu Nepal; ^2^ Patan Academy of Health Sciences Oxford University Clinical Research Unit Lalitpur Nepal; ^3^ Department of Medicine Shree Birendra Hospital Chhauni Kathmandu Nepal; ^4^ Nepalese Army Institute of Health Sciences Kathmandu Nepal; ^5^ Department of emergency medicine Palpa hospital Tansen Nepal; ^6^ Norvic International Hospital Thapathali Kathmandu Nepal

**Keywords:** Behcet disease, Behcet's disease, Behcet's Syndrome, Nepal, orogenital ulcer

## Abstract

This case report highlights considering Behcet's disease as a diagnosis in orogenital ulcers and uveitis, although its prevalence is unknown in Nepal due to underreporting. Also, collaboration for patient care among relevant specialties is required.

## Graphical abstract

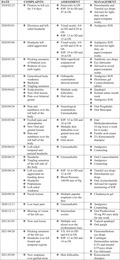

## 1 INTRODUCTION

Behcet's disease is a rare systemic vasculitis characterized by recurrent episodes of acute inflammation affecting blood vessels of all sizes. Symptoms include orogenital apthosis, cutaneous skin lesions, and uveitis. We present the case of a 38‐year‐old Nepalese man with Behcet's disease. In Nepal, Behcet's disease may still be under‐reported.

Behcet's disease is a rare systemic vasculitis with relapsing and remitting episodes of acute inflammation involving all sizes and types of vessels, with more involvement of veins more than arteries.^1^ It usually presents with orogenital apthosis, cutaneous skin lesions, hypopyon, and uveitis,^2^, ^3^ but frequent involvement of the articular system, central nervous system, and gastrointestinal tracts has also been reported.^4^ The usual age of onset is on their third decades of life, and males are more severely affected than females. The disease is more prevalent in Turkish (14–20/100 000),^5^ Mediterranean, Middle East regions, sometimes referred to as the Silk Road disease.^6^ It is less frequent in other parts of the world, including the Indian subcontinent.^7^ The disease is a rare presentation in Nepal, and its prevalence in Nepal is yet to be determined.^4^ Here, we present a case of a 38‐year‐old male patient from Nepal with features of Behcet's syndrome.

## 2 CASE REPORT

About one and a half years ago, a 38‐year‐old married male Nepalese serving soldier presented to our center with complaints of diminution of vision of the left eye for three days. His visual acuities were 6/24 in OS and 6/6 in OD. Intraocular pressure (IOP) was 13 and 12 in OS and OD, respectively. On ophthalmological examination of the left eye, 4+ cells, 2+ flare, blue dot cataract, 3+ vitreous cells, and snow banks were noted. In addition, there were multiple lesions with arteritis in the fundus. The findings of the right eye were unremarkable. There were no abnormalities on systemic examination. With the diagnosis of uveitis (anterior and intermediate), the patient was prescribed prednisolone acetate (1% w/v) 1 drop topically two hourly, and atropine eye drops (1% w/v) TDS.

On further inquiry, he had a history of recurrent episodes of oral ulceration for several months that was aggravated for the last one week. His past medical history revealed the episodic occurrence of genital ulcers a few years back. So, for detailed evaluation, the patient was referred to Dermatology outpatient department (OPD), where a panel of investigations was sent. His routine blood investigations were normal.

Differential diagnoses included aphthous ulcer, secondary syphilis, oro‐mucosal lichen planus, psoriasis vulgaris, systemic lupus erythematosus, and Behcet's disease. To rule out these differential diagnoses, further investigations were sent. Venereal disease research laboratory test, Treponema pallidum hemagglutination test, antinuclear antibody (ANA), anti‐double stranded DNA (anti‐dsDNA) test, human leukocyte antigen (HLA) B27, and HLA B25 came out to be negative. However, he was positive for HLA B51.

Pathergy test was done, which showed a positive result. According to International Criteria for Behcet Disease (ICBD) criteria, all these features were diagnostic of Behcet disease. The patient was then started on oral Methotrexate 15 mg once a week along with oral folic acid 5 mg once weekly. Likewise, the oral ulcer was symptomatically treated with oral saline gargle, quadrajel (Lidocaine, Chlorhexidine Gluconate, and Metronidazole), oroheal gel (Triamcinolone Acetonide 0.1%w/w), zinc, and vitamin C supplements. Since the diagnosis, the patient has been on follow‐up regularly with different complaints at different times, as mentioned in Table 1.

**TABLE 1:** From diagnosis until the present, the clinical presentation and management of a Behcet's disease

Date	Complaints	Assessment	Management
2020/02/23	Floaters in left eye for 3–4 days	Panuveitis in OS IOP: 18 in OD and 47 in OS	Dorzolamide and Timolol eye drops Advised for light‐duty and no paper/computer works
2020/03/01	Dizziness and left‐sided headache	Visual acuity: 6/6 in OD and 6/24 in OS IOP: 12 in OD and 13 in OS	Analgesics SOS
2020/03/06	Headache left‐sided aggravated	Visual acuity: 6/6 in OD and 6/36 in OS IOP: 19 in OD and 18 in OS	Analgesics SOS Advised for light‐duty, no paper/computer works
2020/03/20	Pricking sensation of bilateral eyes Redness of eyes (left>right)	Mild superficial conjunctival congestion	Antibiotic eye drops Eye lubricants Advised to avoid paper/computer works
2020/03/22	Generalized body weakness Backache Tingling sensation	Orthopedic examination showed normal	Analgesics SOS Diclofenac gel Cyanocobalamin tablets
2020/04/02	Scalp pruritus New Oral lesions Pain over bilateral groins	Multiple scalp folliculitis Oral ulcers	Quadrajel Normal saline mouth wash Analgesics SOS
2020/04/28	Pain and numbness over the left half of the body	Neurological examination: Unremarkable	Oral Pregabalin Oral Buscopan
2020/05/06	Eyeball pain and photophobia New Oral and genital lesions Pain and numbness over the left half of the body	IOP: 15 in OD and 14 in OS Multiple skin folliculitis over genital area and scalp Oral ulcers	Oral Methylprednisolone 16 mg twice a week for 4 weeks Fusidic acid cream 2% Ketoconazole shampoo
2020/06/03	Left‐sided temporal and parietal headache	Unremarkable	Analgesics Counseling
2020/06/25	Headache Tingling sensation of the left side of the body	Unremarkable	Oral Cyanocobalmin Analgesics Counseling
2020/08/24	Left eye pain aggravated on bending down Headache Palpitations Left‐sided weakness	IOP: 16 in OD and 32 in OS Blood Pressure: 140/90 mm of Hg	Timolol eye drops Dorzolamide eye drops Oral Acetazolamide Advised for regular monitoring of IOP
2020/09/30	Facial lesions	Multiple papular eruptions over the face	Clindamycin gel
2020/12/13	Low back pain	Unremarkable	Analgesics Counseling
2020/12/13	Blurring of vision of the left eye	Intermediate uveitis	Oral Prednisolone 50 mg PO once daily for one week
2021/01/05	New oral lesion	Multiple oral ulcers over the erythematous base	Topical quadrajel Oral gargle
2021/04/20	Pricking sensation of the left eye Headache over left frontal and temporal regions	VA: 6/6 in OD and 6/6 in OS IOP: 12 in OD and 14 in OS	Fluorometholone eye drops Combigan (brimonidine tartrate 0.2% and timolol 0.5%)eye drops Eye lubricants
2021/05/09	New eruptions over genital areas	Skin folliculitis	Ketoconazole shampoo

On 2021/06/25, the patient presented with complaints of right‐sided chest pain, headache, and dizziness. Vitals were within normal limits. General and systemic examinations were unremarkable. Baseline investigations, including complete blood count, renal function test, liver function test, serum electrolytes, urine routine and microscopic, and chest X‐ray, showed normal findings. Creatinine phosphokinase (CPK‐MB) was 18 IU/L, and Troponin‐I was negative. C‐reactive protein was positive (63.18 mg/L), and erythrocyte sedimentation rate was 40 mm/hr, possibly indicating the active stage of the disease. After that, a non‐contrast CT head and high‐resolution CT chest were done, which were normal. Ear, nose, and throat (ENT) consultation was done for dizziness, but they suggested no possible middle ear causes. His headache was associated with throbbing eyeball pain. On ophthalmologic consultation, peripheral choroiditis and vitritis were noted with normal intraocular pressure and visual acuity. Oral prednisolone 50 mg once daily was started and tapered over several days. Likewise, atropine and prednisolone eye drops were also prescribed. During the hospital stay, the visual acuity deteriorated, and IOP also increased. Oral acetazolamide was started, and IOP gradually decreased to the normal range. Visual acuity was not significantly improved till discharge.

The patient also developed pain and tingling sensation over the left half of the head, face, and neck. In between, he was referred to a rheumatologist, who recommended the use of adalimumab. Sputum smear for acid‐fast bacilli, Mantoux test, and chest X‐ray were done to rule out tuberculosis. Similarly, liver function tests, viral markers, and ultrasound abdomen were performed to rule out viral hepatitis. Finally, the first and second dose of adalimumab 40mg subcutaneously on an interval of fifteen days was administered, and the patient was discharged and advised to follow‐up after two weeks. His ocular symptoms have improved on follow‐up examination.

## 3 DISCUSSION

Behcet's disease (BD) is a rare, systemic disorder initially described by Hulusi Behcet, a Turkish dermatologist, as a triad of uveitis and recurrent oral and genital ulcers. It is prevalent in people of Mediterranean and Middle East countries and less frequent in the Indian subcontinent. The disease usually manifests during the 3rd and 4th decade of their life with male predominance.^8^, ^9^ We present a case report of a male serving soldier from Nepal in his fourth decade of life. Testosterone may play a role in neutrophil and Th‐1 cell activation. This could explain why male patients have a more severe case of BD.^10^

The disease raises the mortality rate, particularly in young male patients. Large vessel involvement (pulmonary artery aneurysm), neurological involvement, gastrointestinal system involvement, and cardiac involvement are the most common causes of mortality.^11^ The exact cause of Behcet's disease is unknown and is thought to be multifactorial. The MHC class I region, which includes HLA‐B*51, contains the strongest genetic risk factor for BD. There is a 5. Seventy eight‐fold higher chance of getting BD for individuals with the HLA‐B*51/B5 allele than those who did not have this gene.^12^ Other potential factors can be microbial factors as oral aphthous ulcer typically precede the systemic presentations and occurs before every recurrence of the disease. This case was positive for HLA‐B51, which demonstrated probable genetic cause for the occurrence of the disease. Although the significance of HLA‐B*51 is well established, it is found to be positive in roughly 60% of patients with Bechet disease. HLA‐B*51's role in the genetic predisposition to the Behcet disease is around 12%–19%.^13^

There is no confirmatory test for diagnosing Behcet's disease as the history and clinical picture are often sufficient for the diagnosis. However, diagnostic criteria proposed by an International Study Group are used for research purposes and clinical purposes too. According to International Study Group criteria for Behcet's disease,^14^ there must be recurrent oral ulcerations (minor aphthous, major aphthous, or herpetiform ulcerations, which recurred at least three times in 12 month period). In addition, two of the following criteria must be met: Recurrent genital ulcerations, eye lesions (Uveitis, cells in vitreous on slit‐lamp examination, or retinal vasculitis), skin lesions (erythema nodosum, pseudofolliculitis, and papulopustular lesions), and positive pathergy test.

The International Criteria for Behcet's disease(ICBD)^15^ are proposed to assist earlier diagnosis as ISG clinical diagnosis has low sensitivity. According to signs and symptoms, the International Criteria for Behcet's disease have a scoring system, two points each for ocular lesions, genital apthosis, and oral apthosis. Each point for skin lesions, neurological manifestations, and vascular manifestations, and positive pathergy test. A score of more than or equals to 4 indicates Behcet's diagnosis. Our patient had ocular lesions, genital apthosis, oral apthosis, skin lesions, vascular manifestations (fundal arteritis), and a positive pathergy test; hence, ICBD score was calculated to be eight, which is strongly suggestive of Behcet's disease.

BD has a relapsing and remitting course; our patient also has relapsing courses of orogenital ulcers, uveitis, and neurological symptoms over 18 months. Uveitis in BD responds well to steroids as our patient's uveitis improved on prednisolone acetate. When vital organs are affected, a combination of corticosteroids and immunosuppressant medications is recommended. Due to relapsing ocular symptoms, he was placed on adalimumab (TNF‐α antagonist). TNF‐α, a pro‐inflammatory cytokine, is involved in the autoimmune response, inflammation induction, and maintenance. Therefore, it becomes a crucial target molecule in the disease's treatment.^16^ Adalimumab was linked to a reduced risk of uveitis aggravation or visual impairment in non‐infectious active intermediate, posterior uveitis, and panuveitis in a placebo‐controlled phase 3 research involving patients with BD.^17^

## 4 CONCLUSIONS

The prevalence of Behcet's disease is unknown in Nepal and the Indian subcontinent. Despite that, it should be considered a differential diagnosis in recurrent orogenital ulcers, as Behcet's disease may still be under‐reported in Nepal. Earlier diagnosis will help delay the progression of the disease and prevent other complications. Regular follow‐up and proper care of BD are required because of the high frequency of vital organ involvement. Collaboration among relevant specialties such as dermatologist, ophthalmologist, internal medicine, neurologist, dentist, and rheumatologist is required to improve patient outcomes due to its multisystemic nature.

## CONFLICTS OF INTERESTS

Authors have no conflicts of interest to declare.

## AUTHOR CONTRIBUTIONS

Madan Basnet and Kamal Gautam wrote the original manuscript. Suman Gaire, Narayan Bohora, and Ayushi Srivastava reviewed and edited the original manuscript. Bishnu Deep Pathak and Abisha Phudong were involved in the management of the case, reviewed and edited the manuscript.

## ETHICAL APPROVAL

Need for ethical approval waived. Consent from the patient's parents deemed to be enough.

## CONSENT

Written informed consent was taken from the patient before writing the manuscript.

## Data Availability

All the necessary data are available in the article itself.
